# Smart financial incentives to promote cardiovascular health

**Published:** 2020-01-16

**Authors:** Donald S Shepard

**Affiliations:** Heller School for Social Policy and Management, Brandeis University, USA

**Keywords:** clinical medicine, smart financial incentives, cardiovascular, diabetes, hypertension, elevated lipids

## Opinion

The United States, like most countries around the world, is striving to improve its population’s cardiovascular health. Recent results, however, are mixed. On the favorable side, clinical medicine continues to improve. For example, a December 2019 review chronicled 39 improvements in the diagnosis and management of cardiovascular disease just in the preceding six months.^1^ On the worrying side, however, several risk factors due to lifestyle continue to worsen. The National Health and Examination Survey (NHANES) documented significant increases in the prevalence of obesity from 1999-2000 to 2015-16 (the most recent data) in both adults and youth.^2^ More recent self-reported data through 2018 showed the prevalence of both obesity^3^ and sedentary lifestyles has continued to worsen.^4^ Obesity increases risk not only for cardiovascular disease, but also for many types of cancer.^5^

The challenge for improving population health is implementing and sustaining approaches to halt, and then reverse these disturbing trends. Accumulating evidence suggests that smart financial incentives offer a promising approach. Several randomized trials have shown how financial incentives can improve cardiovascular indicators. For example, a 16-week trial evaluated both a deposit contract (where participants deposited money, matched by the program that could return up to $252 per month) and a lottery program (daily chances of $10 or $100) based on daily weight compared to daily monitoring without incentives. Participants in the both incentive conditions had significantly greater odds of meeting the program goal of losing one pound per week than control participants. The odds ratios were 7.7; (95% CI, 1.4-42.7) for the deposit approach and 9.4 (95% CI, 1.7-52.7) for the lottery.^6^ A subsequent one-year randomized trial with weekly incentives or penalties of $20 found a highly significant 6.5 pound reduction (standard error 1.92, p<0.001) in the intervention group compared to controls.^7^ A multi-site trial of financial incentives targeted lipid reductions comparing incentives to physicians, patients or both against usual care.^8^ Only the shared incentives between physicians and patients achieved significant improvements over the control group (p=0.002), obtaining an improvement about one third larger than occurred in the control group alone.

**Figure 1:**
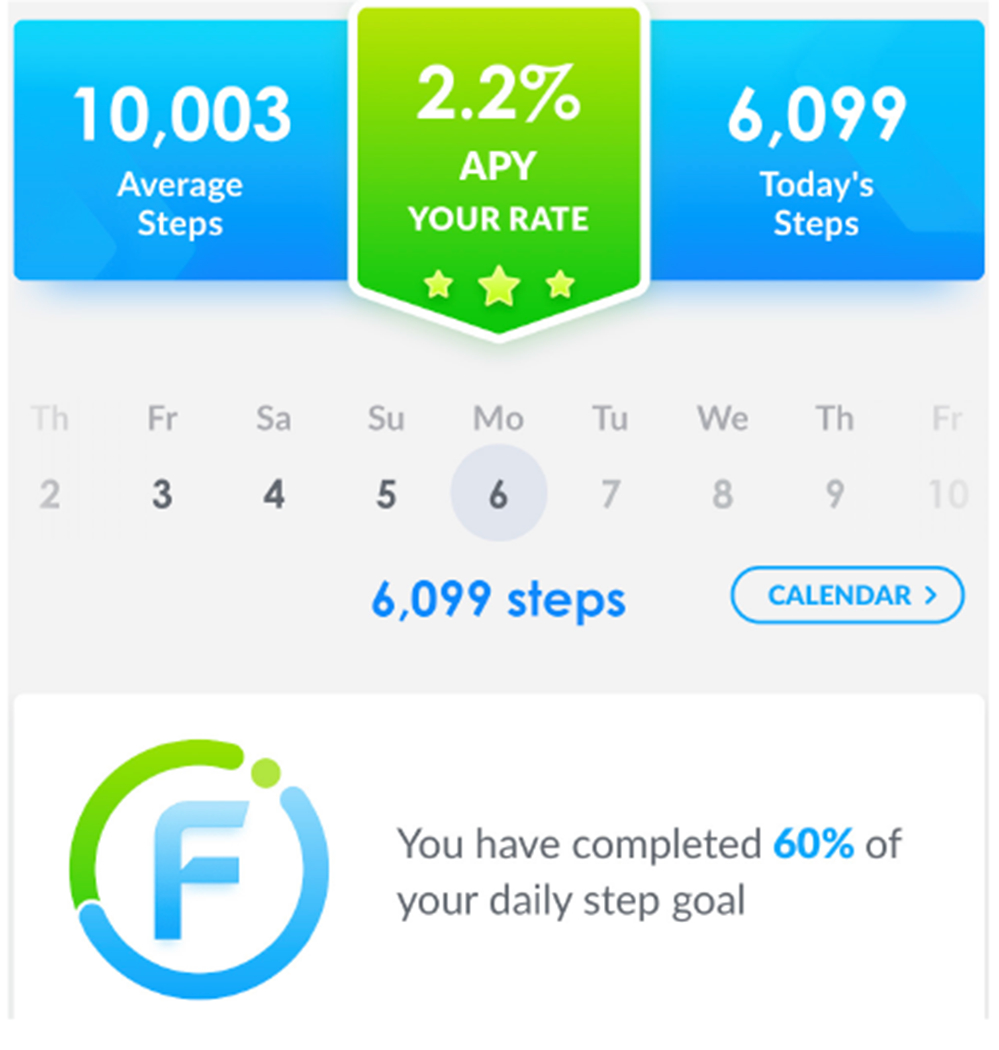
Tiered rates for seniors at FitnessBank.

Outside the area of controlled research, many commercial entitles are establishing incentive programs for customers and workers. Digital options are creating promising new options. The online bank called FitnessBank offers a particularly innovative approach.^9^ The interest rate that an account holder receives each month depends on his/her average daily number of steps in the preceding month. The bank provides a standard rate schedule for working age adults and an easier, senior schedule, for savers age 65 and above. A senior account holder can earn the current maximum annual interest rate (2.2% in December 2019) by documenting a daily average of 10,000 or more steps in the preceding month (Figure 1). By contrast, the highest yielding general online account then paid only 1.85%.^10^

**Figure 2:**
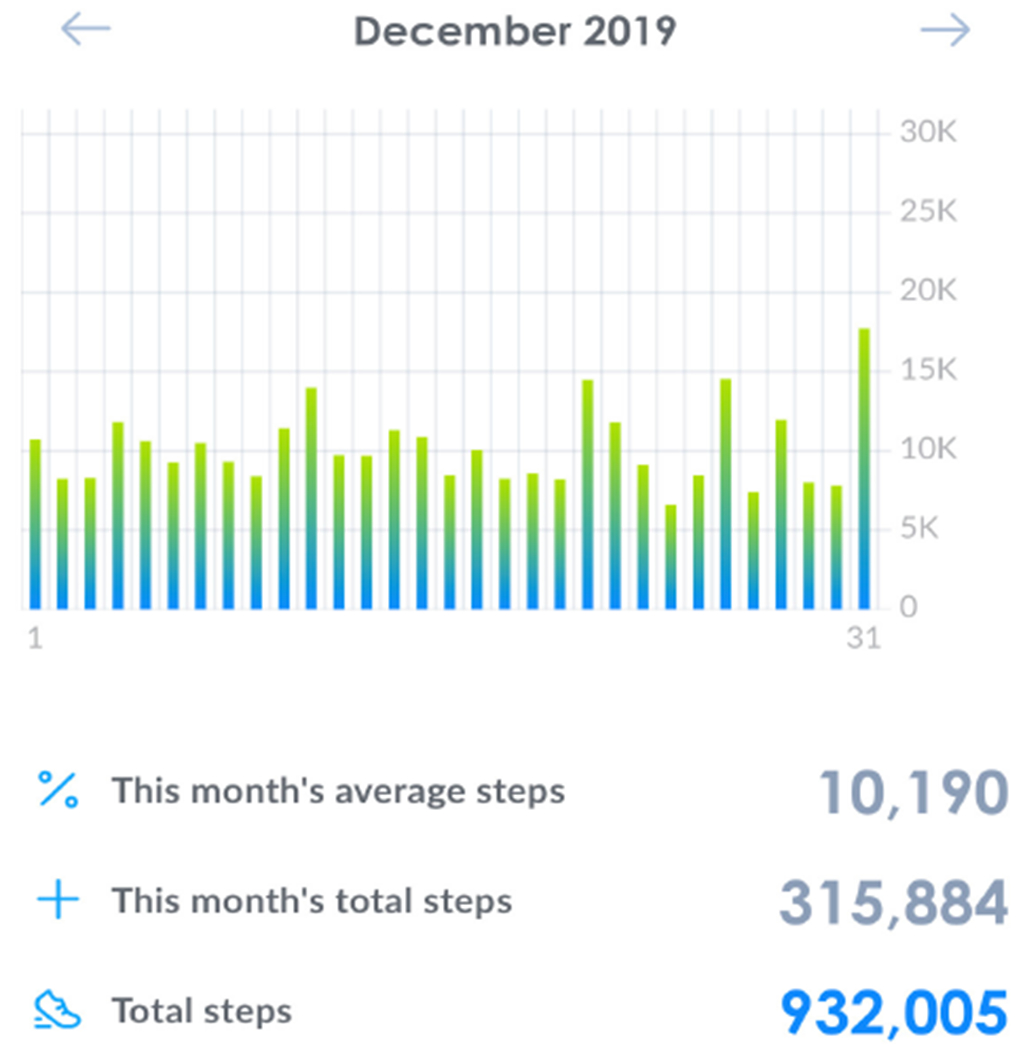
Step Tracker display of monthly statistics.

To access FitnessBank’s tiered rates, the saver must install the bank’s Step Tracker app his/her phone and authorize it to access data from Apple “Health,” Google “Play,” or a similar app. These apps obtain the steps from a smart watch, Bluetooth-enabled wrist band (available for as little as $10), or a pedometer built into late model mobile phones. The Step Tracker automatically retrieves each day’s steps, allows the user to monitor his/her progress (Figure 2), and transfers the monthly average to the bank. The smart feature of this system is that it requires no ongoing user action after setting up Step Tracker. This process avoids both the potential errors of self-reported data and the inconvenience of regular visits to a gym, clinic or laboratory. The bank’s tiered interest rates provide ongoing health motivation for the customer and a source of new customers and referrals for the bank.

In the future, smart digital systems could improve cardiovascular and cancer health through many types of reminders and incentives. Smart pill bottles can remind users to take prescribed medicines and record when the bottle is opened. Smart bathroom scales can record, time stamp, and transmit weight and body fat. Electronic blood pressure cuffs can monitor blood pressure and help clinicians adjust medications. Continuous glucose monitors can help diabetics monitor blood sugar.

Many types of organizations benefit from healthy members and could gain by implementing such incentive plans. Potential implementers include employers, medical providers, health plans, and health, life, and disability insurers. These organizations would realize the greatest gains from members for whom the scope for improvement is large. This would apply in persons currently at elevated risk due to higher age, relevant co-morbidity (e.g. diabetes, hypertension, elevated lipids), other risk factors (e.g. smoking, alcohol abuse, or obesity), past high medical costs, and sedentary lifestyles.

Digital health systems could facilitate such targeted enrollment by collaborating with medical providers or health plans that already possess electronic health information. The providers and plans could ask their members whether they were interested in exploring their eligibility for such a plan. Those interested would allow relevant data to be passed through an algorithm anonymously and told the results. Those eligible would be invited to enroll.

To complement incentives, disincentives or taxes for unhealthy products or behaviors also deserve attention. They have the advantages of applying to the entire population, rather than just volunteers, and generating revenues that can be directed to other meritorious needs. Taxes on tobacco products, the oldest example, were rated by the World Health Organization as “the single most effective way to decrease consumption and encourage tobacco users to quit.”^11^ More recently, some jurisdictions have imposed taxes on sugar sweetened beverages. Mexico’s 2014 national tax of 1 peso (US$0.077) per liter reduced consumption of taxed beverages by 7.6% over the ensuing two years.^12^ The City of Berkeley, California enacted a tax on such beverages in November, 2014. Follow up surveys from 2015 through 2017 in matched low-income diverse neighborhoods in Berkeley and nearby comparison cities (Oakland and San Francisco) also showed significant declines. Average daily consumption of such beverages in Berkeley fell by 0.55 (95% CI = 0.35-0.75) from a baseline 1.25, while comparison cities saw no significant change.

An evaluation of Philadelphia’s “soda tax,” which added about $1.01 (about 65%) to the price of a 2-liter bottle, also showed significant reductions in soda consumption. After adjustment for purchases outside the city limits, calorie consumption declined with borderline significance, with a net change of −16% (95% CI = −33%, 1%). However, the evaluators raised the concern that the financial burden of such taxes may fall disproportionately on lower socioeconomic consumers.^13^ On the other hand, as such consumers tend to have higher rates of obesity, they also have the greatest potential health gains and could be targeted by programs from the additional revenues. As expected, the beverage industry has campaigned heavily against such taxes.^14^

## Conclusion

In conclusion, financial incentives (implemented by insurers and similar organizations) and taxes and penalties (enacted by national or local governments) both appear to be promising approaches to promoting cardiovascular health.
